# Tumor patterns and cancer risk in carriers of *TP53* exonic germline variants that alter mRNA splicing

**DOI:** 10.1038/s41431-026-02061-6

**Published:** 2026-03-03

**Authors:** Deborah Schönegger, Emilie Montellier, Sandrine Blanchet, Claire Freycon, Paola Monti, Catherine Goudie, Gaëlle Bougeard, Christian P. Kratz, Pierre Hainaut, Anna Reymer

**Affiliations:** 1 University Grenoble Alpes, Inserm 1209, CNRS 5309, Institute for Advanced Biosciences, Grenoble, France; 2 Department of Pediatric Hematology-Oncology, Grenoble Alpes University Hospital, Grenoble, France; 3 Neuro-oncology and Mutagenesis, IRCCS Azienda Ospedaliera Metropolitana, Genoa, Italy; 4 Department of Pediatrics, Division of Hematology-Oncology, Montreal Children’s Hospital, McGill University Health Centre, Montreal, QC Canada; 5 University Rouen Normandie, Inserm U1245, Normandie Univ, CHU Rouen, Department of Genetics, Rouen, France; 6 Pediatric Hematology and Oncology, Hannover Medical School, Hannover, Germany

**Keywords:** Cancer genomics, Risk factors, Transcriptomics

## Abstract

Abnormal RNA splicing is an underrecognized driver of pathogenicity in germline *TP53 —* the cause of Li–Fraumeni syndrome (LFS). We re-evaluated exonic single-nucleotide variants (SNVs) that yield missense or synonymous changes for spliceogenic effects by integrating SpliceAI prediction, in-vitro minigene assays, and analysis of tumor RNA-seq from TCGA, and assessed genotype-phenotype correlations using clinical data from multiple databases and national registries. We identified 58 spliceogenic exonic SNVs (SE-SNVs) across the *TP53* gene (40 missense, 18 synonymous). Experimental validation confirmed aberrant splicing for 15 out of 17 tested variants, most often through cryptic splice-site activation that introduced frameshifts and premature termination. Clinically, carriers of SE-SNVs previously considered as mild or of low-pathogenicity by protein-based assays showed earlier onset and LFS-signature cancers, indicating that splicing disruption can override amino-acid effects. The recurrent c.375 G > A (p.(Thr125 = )) showed heterogeneous effect: with both childhood/adolescent and adult onset, consistent with partial, variable retention of canonical splicing. These data reveal a substantial burden of spliceogenic pathogenicity in *TP53* and strong support integrating splicing prediction, functional validation, and transcript-level evidence into variant interpretation and risk stratification in LFS.

## Introduction

Abnormal RNA splicing contributes to gene inactivation in cancer-predisposition syndromes, including Li–Fraumeni syndrome (LFS), a heterogeneous autosomal dominant predisposition to various cancers caused by pathogenic germline *TP53* variants [1, 2]. While most reported LFS variants are exonic single-nucleotide variations (SNVs) that alter amino acids, about 10% affect intron–exon boundaries or intronic regulatory motifs and disrupt splicing; moreover, exonic SNVs themselves can be spliceogenic, altering mRNA processing in addition or instead to their protein-coding effect [3–9]. Such spliceogenic exonic SNVs (SE-SNVs) can activate otherwise cryptic splice sites, leading to exon skipping or intron retention often resulting in nonsense-mediated decay (NMD) or, in some instances, stable but incorrectly spliced RNA that produces a functionally impaired protein [6, 7]. Since splicing alterations can override amino-acid substitutions, SE-SNVs may be misclassified when only protein structure/function is considered.

LFS confers lifelong, multi-cancer risk with age-specific peaks in childhood, adolescence, and adulthood, producing a complex tumor spectrum that is hard to predict at individual or family level [10, 11]. Clinical and functional studies show robust links between *TP53* variant structure, in vitro functional activity, age at onset, and tumor profiles in carriers [5, 12–14]. We recently proposed a four-tier classification of *TP53* missense variants (Classes A–D) capturing a gradient from very severe LFS (A), through intermediate forms (B, C), to variants with infrequent “LFS-signature” tumors (D) [5]. Notably, this scheme was built on the yeast transactivation dataset of Ishioka and colleagues [15] and therefore does not incorporate potential spliceogenic effects of the underlying exonic SNVs.

Recent advances now enable finer assessment of SE-SNVs. First, Stiewe and collaborators have developed a saturation genome-editing screen that introduced SNVs into the endogenous *TP53* locus (introns and exons) to compare variant effects on p53 function in a standardized, physiologic context [9]. Second, Spurdle and colleagues established an integrated in silico prediction–minigene validation framework, which they applied to 59 *TP53* candidates to delineate the spectrum and magnitude of splicing defects and provide pathogenicity-relevant evidence [16]. Together, these tools allow locus-native functional readouts alongside splicing-specific validation, sharpening phenotypic inference for *TP53* SE-SNVs.

Building on these advances, we re-assessed all missense variants in our functional classification [5] for spliceogenicity of the underlying SNVs by integrating bioinformatic prediction, in vitro minigene reporter assays, and analysis of tumor RNA-seq data from The Cancer Genome Atlas (TCGA) program. We then analyzed genotype-phenotype correlations in germline carriers drawn from the IARC/NCI database [17], supplemented with additional curated cases from the literature (systematic review by C. Freycon, unpublished; see Supplementary Table 1), and the French [2] and German [18] LFS registries, with no overlap with IARC/NCI. This analysis identified several pathogenic SE-SNVs that generate missense variants previously classified as Class C/D by protein-based assays, in which splicing disruption overrides the protein-coding effect. Our study provides the first systematic evaluation of LFS phenotypes in carriers of *TP53* SE-SNVs that yield missense or silent protein changes, clarifying their molecular basis and clinical impact.

## Results

### SpliceAI predictions of *TP53* SNVs

**Fig. 1:**
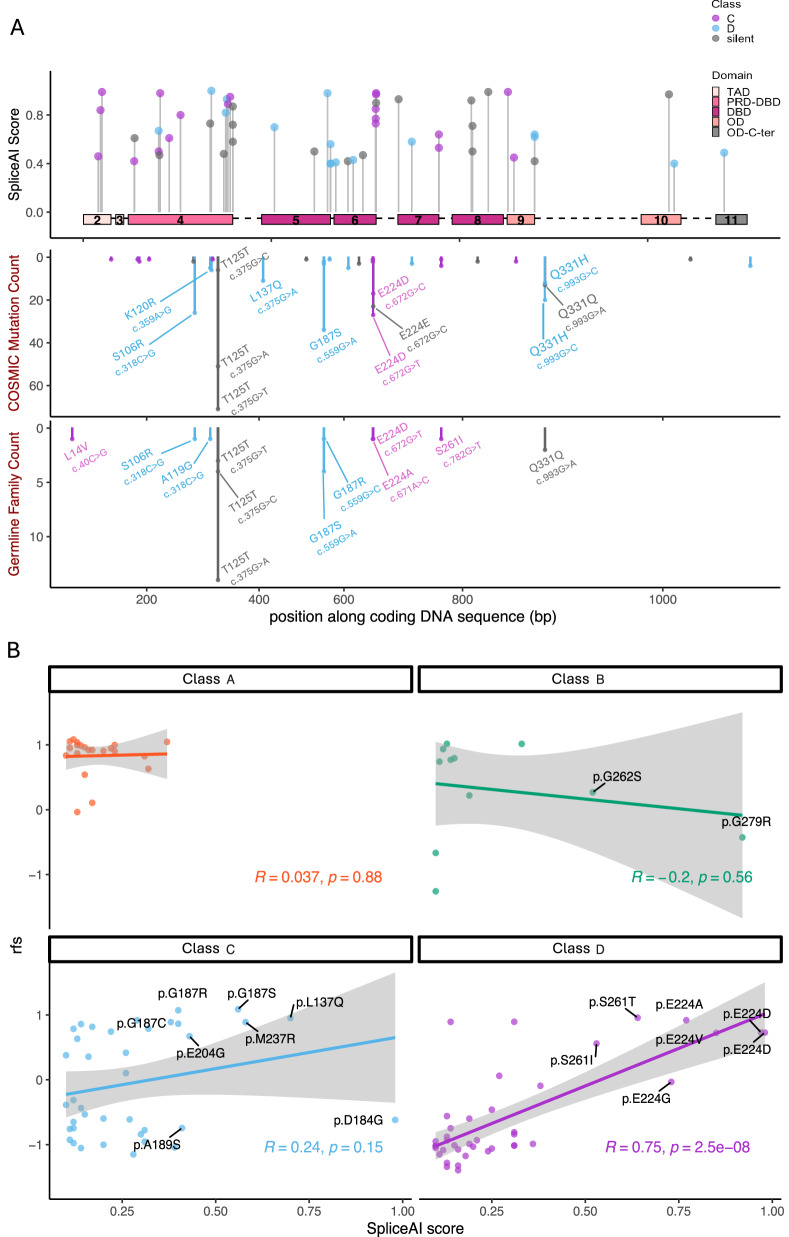
Distribution of spliceogenic exonic single nucleotide variations (SE-SNVs) in the *TP53* gene and relationships with missense variant functional classes. **A** Top panel: SpliceAI delta scores versus location of SE-SNVs (Class C: blue; D: violet; synonymous: grey) variants along the *TP53* coding sequence (threshold: SpliceAI ≥0.4, see text). Exons are represented as boxes labeled by exon number and colored according to the protein domain, introns (not to size) are outlined as dotted lines. Lower panels: Distribution of SE-SNVs among COSMIC (middle panel) and germline (bottom panel) *TP53* datasets (see text). **B** Correlation between the relative fitness score (rfs) [9], and SpliceAI delta scores of SE-SNVs, stratified by *TP53* functional class [5]. Red: Class A (very severe variants, including *TP53* hotspot variants, recapitulating all severe traits of LFS); Green: Class B (severe variants); Blue: Class C (heterogeneous variants associated with less severe LFS traits); Violet: Class D (mostly predicted benign variants not associated with typical LFS cancer spectrum). Rfs ranges from -1 (functional, wild-type reference) to +1 (non-functional, similar to nonsense mutations). Variants with SpliceAI delta scores ≥0.4 are labeled by their protein change.

We assessed the splicing impact of *TP53* exonic SNVs (exons 2–11) with SpliceAI, a deep neural network tool that predicts splice junctions and splice-altering effects from pre-mRNA sequence [19]. Spliceogenic exonic SNVs (SE-SNVs) were defined as variants with SpliceAI delta score ≥0.4, a threshold balancing sensitivity (97.9%) and specificity (72.4%) (AUC = 0.95; Supplementary Fig. 1). Candidate SE-SNVs, otherwise yielding missense or synonymous changes, were predicted across the coding region with exon-specific density differences (Fig. 1A). In total, 58 SE-SNVs were identified (Supplementary Table 2): 40 missense and 18 synonymous. Forty-two appeared in germline or somatic T*P53* databases, representing a small fraction of entries (COSMIC: 40/1761, 2.3%; IARC/NCI *TP53* somatic: 58/3409, 1.7%; *TP53* germline: 13/314, 4.1%; gnomAD: 12/730, 1.6%). Frequently represented were synonymous substitutions at codons 125 (c.375 G > T, c.375 G > A, c.375 G > C; p.(Thr125 = )), 224 (c.672 G > C; p.(Glu224 = )), and 331 (c.993 G > A; p.(Gln331 = )), and missense substitutions at 106 (c.318 C > G; p.(Ser106Arg)), 119 (c.356 C > G; p.(Ala119Gly)), 120 (c.359 A > G; p.(Lys120Arg)), 187 (c.559 G > A; p.(Gly187Ser); c.559 G > C; p.(Gly187Arg)), 224 (c.672 G > C, c.672 G > T; p.(Glu224Asp)), and 331 (c.993 G > T, c.993 G > C; p.(Gln331His)). The same SNVs tended to occur in somatic and germline datasets (Fig. 1A; Supplementary Table 2), implying relevance in both contexts, but their distribution was more restricted in the germline. In the germline dataset, c.375 G > A (p.(Thr125 = )) accounted for 38% (14 families, 59 cases), followed by c.559 G > A (p.(Gly187Ser), 11%), c.375 G > T (p.(Thr125 = ), 11%), c.375 G > C (p.(Thr125 = ), 8%), and c.993 G > A (p.(Gln331 = ), 7%); no other variant exceeded 3%. In somatic (COSMIC) data, the most frequent were c.375 G > T (p.(Thr125 = ), 17%), c.375 G > A (p.(Thr125 = ), 13%), c.559 G > A (p.(Gly187Ser), 8%), c.672 G > T (p.(Glu224Asp), 7%), c.318 C > G (p.(Ser106Arg), 6%), c.672 G > A (p.(Glu224 = ), 6%), and c.993 G > T (p.(Gln331His), 5%); remaining variants were each <5% (Supplementary Table 2).

Missense variants were annotated into functional Classes A–D proposed by Hainaut and collaborators [5], representing a gradient of transcriptional loss in a standardized in-vitro assay that correlates with clinical severity in germline *TP53* carriers: from Class A (most severe; Pathogenic/Likely Pathogenic) to Class D (near–wild-type; Benign/Likely Benign). Candidate SE-SNVs were largely identified among Classes C/D and synonymous variants (91.4%; Fig. 1A; Supplementary Table 2), whereas only 8.6% of predicted SE-SNVs fell in Classes A/B (Supplementary Table 2).

To assess the functional impact of these SE-SNVs according to their corresponding missense variant class, we analyzed the correlation between SpliceAI delta scores and the relative fitness scores in the CRISPR-mediated homology-directed repair assay developed by Stiewe and collaborators [9] (Fig. 1B, Supplementary Table 3). The fitness scores (rfs) vary from -1 (i.e*.*, variants retaining a quasi-wild-type activity) to +1 (*i.e*., the most severely impaired variants). As expected, Class A and B SE-SNVs tended to have higher rfs values than Class C or D variants, consistent with their more severe structural and functional alterations. For former classes, SpliceAI delta scores showed no correlation with rfs. In contrast, among Class C and especially Class D variants, a positive correlation between the two scores was observed, reaching strong statistical significance in Class D (*p* < 0.001). These findings suggest that several Class C and D variants, classified as attenuated or benign based solely on the functional impact of their amino acid substitutions, may exert pathogenic effects through splicing alterations that override their protein-coding consequences.

### Functional analysis of SE-SNVs

We next verified SpliceAI-predicted defects with a pSPL3-based in vitro minigene assay in COS1 fibroblast-like monkey cells. The exon-trapping vector (two endogenous exons, V1 and V2) accepts genomic inserts at a central cloning site. The *TP53* sequence was split into three fragments: exons 2–4, 5–8, and 8–10 for testing (Supplementary Fig. 2). We assessed Class C/D SE-SNVs with SpliceAI delta score ≥0.75 and synonymous candidates with delta score ≥0.4 for altered splicing versus the empty-vector control (representative readouts in Supplementary Fig. 2). Among the identified 17 SE-SNVs (highlighted in yellow in Supplementary Table 2), 15 (88%) produced aberrant splicing, exon skipping or intron inclusion, through variant-induced altered splice motifs (Table 1; Supplementary Fig. 3). Most events predicted frameshifts and premature stop codons, consistent with nonsense-mediated decay; for several variants, aberrant and canonical transcripts co-existed. Two variants, c.46 C > A (p.(Gln16Lys)) and c.362 C > A (p.(Ser121Tyr)), were negative in this assay, with sequencing showing only canonical splicing and no cryptic-site activation. A distinctive pattern occurred with c.551 A > G (p.(Asp184Gly)): an in-frame deletion of three residues (D184-S185-D186) at the end of exon 5, with partial retention of normal splicing. Notably, this SNV was an outlier in Fig. 1B, showing SpliceAI delta score =1 (highly spliceogenic) but a low rfs ( − 0.62) indicative of quasi–wild-type function; whether the resulting protein retains activity remains unknown.

**Table 1:** Functional and transcript-level characterization of TP53 spliceogenic exonic variants (SE-SNVs).

			Minigene Assay, COS1 cells^1^			TCGA mRNA analysis^2^		Gene Editing^3^	Minigene Assay, SK-BR-3 cells^4^	
Variant (NM_000546.6)	Protein change	SpliceAI	Canonical^5^	Frameshift altered	Inframe altered	Canonical^6^	Altered	rfs	Canonical^7^	Altered
c.46 C > A	p.(Q16K)	0.84	+	–	–	na	na	na	47% ± 0.7%	Δ(E2q31):[53% ± 0.7%]
c.50 A > T	p.(E17V)	0.99	–	Δ(E2q26)	–	na	na	na	–	Δ(E2q26):[92.3% ± 1.3%]
c.318 C > G	p.(S106R)	1	–	–	Δ(E4q58)	Reduced: (-86.1%)	na	na	–	Δ(E4q58):[100%]
c.356 C > G	p.(A119G)	0.82	–	Δ(E4q20), Δ(E4q200)	–	na	na	na	5.1% ± 0.3%	Δ(E4q20):[24.0% ± 2.7%], Δ(E4q200):[70.9% ± 2.7%]
c.359 A > G	p.(K120R)	0.93	–	Δ(E4q16)	–	na	na	na	17.5% ± 0.8%	Δ(E4q16):[67.5% ± 0.3%]
c.362 C > A	p.(S121Y)	0.89	+	–	–	na	na	na	82.8% ± 0.2%	Δ(E4q16):[17.2% ± 0.2%]
c.368 C > G	p.(T123S)	0.95	±	–	Δ(E4q12)	na	na	na	59.9% ± 0.7%	Δ(E4q12):[40.1% ± 0.7%]
c.375 G > A	p.(T125 = )	0.58	±	Δ(E4q200)	–	Reduced: (-63.7%)	▼I4	na	na	na
c.375 G > C	p.(T125 = )	0.87	±	Δ(E4q200)	–	Reduced: (-71.5%)	▼I4	na	na	na
c.375 G > T	p.(T125 = )	0.72	±	Δ(E4q200)	–	Reduced: (-72%)	▼I4, Δ(E4q200)	na	na	na
c.551 A > G	p.(D184G)	0.98	±	–	Δ(E5q9)	na	na	-0.62	37.8% ± 0.6%	Δ(E5q9):[62.2% ± 0.6%]
c.671 A > C	p.(E224A)	0.77	–	▼(E6q5)	–	na	na	0.92	–	▼(E6q5):[87.6% ± 0.2%], Δ(E6):[7.1% ± 0.1%]
c.671 A > T	p.(E224V)	0.85	–	▼(E6q5)	–	na	na	0.72	5.9% ± 0.1%	▼(E6q5);[81.2% ± 0.3%], Δ(E6):[12.9% ± 0.4%]
c.672 G > T	p.(E224D)	0.97	–	▼(E6q5)	–	Reduced: (-76%)	na	0.73	–	▼(E6q5):[95.7% ± 1.7%], Δ(E6):[4.3% ± 1.7%]
c.672 G > A	p.(E224 = )	0.9	–	▼(E6q5)	–	Reduced: (-47.4%)	▼(E6q5)	na	na	na
c.922 C > G	p.(L308V)	0.99	±	–	Δ(E9p3)	na	na	na	–	Δ(E9p3):[100%]
c.993 G > A	p.(Q331 = )	0.43	±	▼(E9q328)	–	Reduced: (-38.8%)	▼(E9q328)	na	na	na

The table lists single nucleotide variants (SNVs), corresponding protein changes, SpliceAI predictions, and observed transcript consequences, including frameshift or in-frame events, for 17 tested SE-SNVs from Class C and D with SpliceAI delta score ≥ 0.75 as well as those resulting in silent variants with SpliceAI delta scores ≥ 0.4. Presented are data generated in the current study: minigene assays in COS1 cells (1) and TCGA mRNA analysis (2); together with published functional results from CRISPR/Cas9 gene editing(3) (Ref. [9]); and minigene assays in SK-BR-3 cells (4)(Ref. [16]). Levels of normally spliced p53 mRNA (“canonical”) were estimated by semi-quantitative sizing of sequencing peaks (5; stratified as +, ±, -), mRNA read counts (6; % of total reads), or semi-quantitative PCR (7; % of total p53 mRNA). Symbols: Δ = exon skipping (or partial skipping); ▼ = intron retention (or partial retention). q/p notation indicates nucleotide position within the exon (q = downstream portion) or intron (p = upstream portion) relative to the splice junction. na = not available.

To gain further insight into how these SE-SNVs affect splicing patterns in vivo, we analyzed public RNA-Seq transcriptomic data from The Cancer Genome Atlas (TCGA). Raw RNA-Seq read alignments were examined to determine the exact structure of SE-SNV–associated splicing events and to compare them with those observed in the minigene assay. Of the 17 SE-SNVs, seven (41.2%) have been identified in solid tumors in the TCGA dataset. Analysis of these cases confirmed strong spliceogenic effects, consistent with the results from the minigene assay (Table 1, Supplementary Fig. 4). For c.375 G > T (p.(Thr125 = )), RNA-Seq reads revealed intron retention in addition to the cryptic splice site activation observed in the minigene system.

We compared our minigene assay and RNA-Seq read alignment results with those of Fortuno et al. [16], who used a conceptually similar minigene assay incorporating *TP53* exons 2–9 but based on a different cell model (SK-BR-3 breast cancer cells) (Table 1). The comparison revealed high concordance between the two datasets. Notably, the SK-BR-3–based assay detected additional events not observed in our COS1-based assay, including: (1) for c.671 A > C (p.(Glu224Ala)), c.671 A > T (p.(Glu224Val)), and c.672 G > T (p.(Glu224Asp)), exon 6 skipping in addition to the retention of five intronic nucleotides also detected by us; and (2) for c.46 C > A (p.(Gln16Lys)) and c.362 C > A p.(Ser121Tyr), skipping of 31 nucleotides in exon 2 (ΔE2q31) and skipping of 16 nucleotides in exon 4 (ΔE4q16), respectively, whereas these SNVs showed only canonical splicing patterns in our analyses.

### Genotype-phenotype correlations

We proceeded with the analysis of age at onset and tumor phenotypes in germline carriers of SE-SNVs identified by SpliceAI, minigene, and transcriptomic analyses, comparing them with carriers of other protein-altering variants. We asked whether SE-SNVs previously classified as Class C/D by protein-based assays confer more severe phenotypes than predicted. We assembled an extended dataset of carriers with SE-SNVs (synonymous or Class C/D variants) by integrating three sources: (i) the public IARC/NCI germline database [17] (version 20, last updated 2025), (ii) additional cases curated in a recent systematic literature review (2019–2025; C. Freycon, unpublished; see Supplementary Table 1), and (iii) non-redundant cases from the French [2] and German [18] LFS clinical registries. This analysis identified 18 carriers of SE-SNVs classified as Class C (c.318 C > G (p.(Ser106Arg)), c.356 C > G (p.(Ala119Gly)), c.559 G > C (p.(Gly187Arg)), c.559 G > A (p.(Gly187Ser))) or Class D (c.672 G > T (p.(Glu224Asp)), c.40 C > G (p.(Leu14Val)), c.671 A > C (p.(Glu224Ala)), c.782 G > T (p.(Ser261Ile))) variants, who collectively developed 23 cancers (diagnosis age range: 2–50 years). For comparison, we examined: (1) carriers of non–SE-SNVs Class A, B, C, or D variants (*n* = 1426, 290, 238, and 171, respectively); (2) carriers of SNVs at intronic splice sites (*n* = 219), included as a reference group of splice-disrupting mutations, since they are either located at canonical splice sites or annotated as experimentally validated to alter splicing in the IARC/NCI database; and (3) carriers of the SE-SNV c.375 G > A (p.(Thr125 = )), the most frequent variant in the germline *TP53* dataset (*n* = 59 carriers who developed 61 cancers) (Supplementary Table 4).

**Fig. 2:**
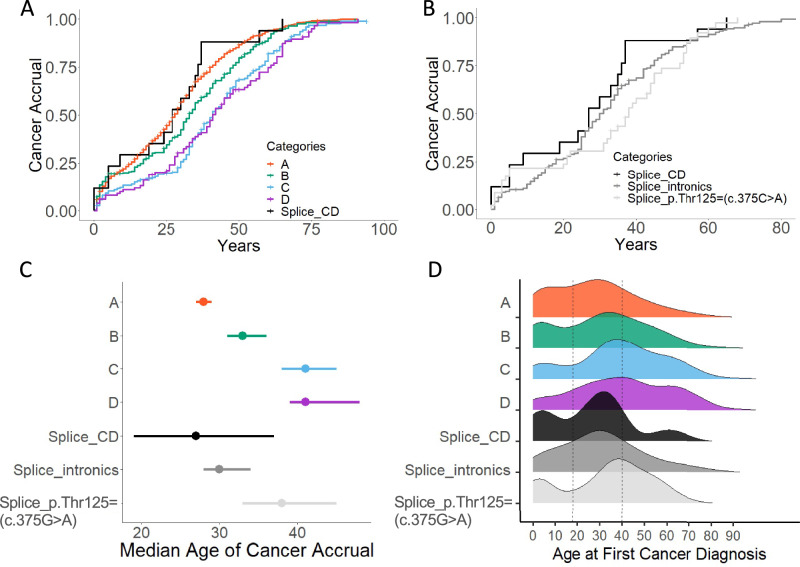
Genotype-phenotype correlations for SE-SNVs among carriers of germline *TP53* variants. **A** Cancer accrual in carriers of SE-SNVs, previously classified as Class C-D variants (black line) compared to non-SE deriving missense variants stratified by functional Class A–D, color-coded as in Fig. 1. **B** Cancer accrual in carriers of SE-SNVs previously classified as Class C-D variants (black line) compared with carriers of intronic spliceogenic SNVs (canonical splice donor/acceptor sites, dark grey line) or of the synonymous SE-SNV c.375 G > A (p.(Thr125 = ); light grey line). **C** Median age at first cancer diagnosis and 95% confidence intervals for each variant group, color coded as above. **D** Density plots showing the age distribution of the first cancer diagnosis for each variant category, as defined and color-coded above.

Results (Fig. 2A–D) showed Class C/D SE-SNV carriers had a median diagnosis age of 27 years, similar to Class A carriers (28 years) and comparable to intronic splice variant carriers (30 years). In contrast, carriers of non–SE-SNV Class C/D variants had a markedly higher median age at diagnosis ( ≥ 40 years). These data suggest that, for Class C/D SE-SNVs, spliceogenic effects may override amino-acid substitution effects. Notably, p.(Thr125 = ) *TP53* variant carriers had a median diagnosis age of 38 years, intermediate between Class B and Class C carriers (Fig. 2D); and showed the bimodal distribution (Fig. 2D) with cases in childhood/adolescence and in adulthood, indicating a wide severity spectrum. The number of carriers (n) for each SNV category analyzed in this study is provided in Supplementary Table 4.

The diagnosis age distribution of first cancers (Fig. 2D) and of all cancers (Supplementary Fig. 5) further supports that Class C/D SE-SNVs confer greater severity than non-spliceogenic counterparts, with a greater proportion of cases arising in childhood and adolescence. Tumors included adrenal cortical carcinoma in childhood, teenage osteosarcoma and astrocytoma, and pre-menopausal breast cancer—all typical of the LFS spectrum (Supplementary Fig. 6); age at onset again showed two peaks (childhood/teenage and early adulthood). The *TP53* c.375 G > A (p.(Thr125 = )) variant carriers similarly exhibited LFS-typical tumors but with onset shifted toward adulthood compared with Class A/B carriers. Among three c.993 G > A (p.(Gln331 = )) carriers, one developed adrenal cortical carcinoma (age 5–10) and two developed breast cancer (age 35–40). Overall, findings support that SE-SNVs can produce more severe phenotypes than predicted from amino-acid changes alone, although limited sample size, especially for Class C/D SE-SNVs, precluded robust statistics.

Together, the 18 carriers of Class C/D SE-SNVs identified here account for 4.8% of all Class C and 3.4% of all Class D germline *TP53* variants in our dataset (Supplementary Table 4). These proportions give an indication of their relative frequency in the LFS setting but should be interpreted with caution, given dataset incompleteness, differences in ascertainment, and the inclusion of additional cases from systematic review and the national registries.

## Discussion

In this study, we combined bioinformatic prediction, minigene reporter assays, and tumor RNA-seq of *TP53* to assess spliceogenic effects of exonic single-nucleotide variants. We confirmed that several spliceogenic exonic SNVs (SE-SNVs), otherwise annotated as synonymous or missense, disrupt canonical splicing, leading to partial or complete exon skipping or intron retention resulting in frameshifts and premature stop codons. Among the identified SE-SNVs in *TP53* germline carriers, clinical severity reflected spliceogenic rather than protein-level effects.

This was most evident for SE-SNVs previously assigned Classes C/D by yeast transactivation assays [5, 15]; affected carriers displayed phenotypes typical of LFS, similar to intronic splice-site carriers and Class A and B missense carriers. These observations indicate splicing effects can override protein-coding effects, supporting reclassification toward more severe categories, consistent with ClinVar Pathogenic/Likely Pathogenic annotations [12]. These genotype-phenotype correlation analyses were constrained by the small size of the SE-SNV carrier dataset and the intrinsic heterogeneity of LFS phenotypes, which preclude robust statistical evaluation. In addition, the analyzed cohorts were ascertained phenotypically, whereas more accurate risk estimates could be achieved through the analysis of genomically ascertained cohorts.

To place our results in the context of the resource most commonly used in clinical genetics, we integrated current ClinVar assertions for all 58 selected SE-SNVs into Supplementary Table 2. ClinVar submissions generally follow the ACMG/AMP five-tier framework (benign, likely benign, VUS/uncertain significance, likely pathogenic, pathogenic), and for *TP53* this is further supported by gene-specific ClinGen specifications (e.g., the *TP53* Variant Curation Expert Panel, VCEP). Among the experimentally tested SE-SNVs, six are currently classified as Pathogenic/Likely Pathogenic in ClinVar: c.375 G > A, c.375 G > C, c.375 G > T (p.(Thr125 = )), c.672 G > A (p.(Glu224 = )), c.993 G > A (p.(Gln331 = )), and c.318 C > G (p.(Ser106Arg)). For some of these, ClinVar submissions explicitly reference spliceogenic loss-of-function evidence (e.g., application of PVS1 (RNA)), but ClinVar entries do not consistently indicate whether splicing evidence contributed to the final assertion across submitters. Therefore, we added a dedicated Supplementary Table 2 column indicating whether ClinVar submissions mention spliceogenicity (prediction-only vs RNA-based evidence, where stated).

Our genotype-phenotype analyses also highlight heterogeneity. In particular, carriers of c.375 G > A (p.(Thr125 = )), the most frequent SE-SNV in LFS, showed variable age at onset and tumor spectra compared with Class A/B and intronic-splice carriers. Of note, c.375 G > A occurs within a methylated CpG site, a mutation-prone position due to spontaneous deamination of 5-methylcytosine to thymine [20]. This unique feature among codons specifying SE-SNVs may explain its frequent occurrence among LFS patients. Variability likely reflects incomplete disruption of canonical splicing, as suggested by low-level normal transcripts in our assays. Since minigene and tumor RNA-seq analyses cannot fully capture physiological complexity, the impact of SE-SNVs probably depends on both aberrant splicing and the degree of residual normal splicing, which may be tissue-specific and modulate pathogenicity.

The recent study by Fortuno et al. [16] systematically explored the role of splicing in *TP53* variant pathogenicity using SpliceAI predictions and minigene assays. The authors found general agreement between prediction and validation, but that the maximum SpliceAI delta score does not quantify aberrant expression. Our results concur; SpliceAI, a deep neural network trained on genome-wide human transcriptome annotations from the GENCODE and GTEx projects, predicts spliceogenic effects by modeling long-range sequence dependencies (up to 10,000 bp) around each position [19, 21]. This algorithm captures both core splice site signals and auxiliary splicing regulatory features, indirectly integrating biological determinants such as RNA secondary structure, chromatin accessibility, and nucleosome positioning that influence splice site recognition.

In our analyses, a SpliceAI delta score ≥0.4 correctly identified all SE-SNVs (resulting in synonymous and missense changes) annotated in either germline (IARC/NCI) or somatic (COSMIC) *TP53* mutation databases. However, SpliceAI also assigned high scores ( ≥ 0.8) to several variants not yet documented in these cancer databases. Among these, SE-SNVs encoding Class D variants such as c.551 A > G (p.(Asp184Gly)), c.46 C > A (p.(Gln16Lys)), and c.368 C > G (p.(Thr123Ser)) showed at least partial retention of normal splicing patterns (40–60%) in our assay. While these findings suggest that residual splicing may suffice to maintain some p53 functionality, quantitative estimates from minigene systems should be interpreted with caution, as they do not fully recapitulate endogenous splicing and require validation in patient-derived samples. In contrast, other SE-SNVs resulting in Class D variants, such as c.50 A > T (p.(Glu17Val)) and c.671 A > C (p.(Glu224Ala)), showed near-complete absence of wild-type splicing patterns in minigene assays and are thus probably pathogenic. In the case of c.671 A > C (p.(Glu224Ala)), its CRISPR-mediated introduction into the *TP53* locus caused near-complete loss of p53 suppressive activity (rfs = 0.92) [9]. Consistent with this predicted severity, this variant was recently identified in a family fulfilling Chompret’s criteria for LFS [22].

Despite overall concordance, our minigene assay results differed from those of Fortuno et al. [16], who detected additional aberrant splicing events. This was particularly evident for SE-SNVs at codon 224 (p.(Glu224Ala), p.(Glu224Val), p.(Glu224Asp)), for which Fortuno et al. [16] reported exon 6 skipping in addition to the retention of five intronic nucleotides also detected in our assay, and for SE-SNVs resulting in p.(Gln16Lys) and p.(Ser121Tyr), which showed altered splicing in their study but only canonical splicing in ours. Methodological factors likely contribute to the observed discrepancies, including their SK-BR-3 (cancer) model versus our COS1 (non-transformed) cells. Given that splicing dysregulation is common in cancers, differences may also reflect somatic versus germline contexts and inherent tissue specificity of splicing, which is a critical consideration when evaluating phenotypic consequences in Li-Fraumeni syndrome. Assay design also differed (single construct spanning exons 2–9 vs three constructs: 2–4, 5–8, 8–10); thus, additional comparative studies will be valuable to determine how different experimental designs influence splicing readouts and to ensure accurate classification of *TP53* variants in germline carriers.

In conclusion, our results underscore that *TP53* missense variants previously classified as benign or likely benign based on protein structural and functional properties can drive severe LFS phenotypes via splice disruption. Although SE-SNVs producing such missense variants are rare in LFS patients, they should not be overlooked, particularly in individuals with borderline or atypical LFS presentations. While SpliceAI predictions offer a reliable first step in detecting potential splice defects, our findings emphasize the need for in-depth functional characterization to assess variant pathogenicity. Such evaluation should include both the identification of aberrantly spliced transcripts and the quantification of residual correctly spliced p53 mRNA, as this is not accurately predicted by SpliceAI and may influence pathogenicity depending on the functional integrity of the encoded p53 protein. Implementing allele-specific transcript analysis in peripheral blood cells, providing both qualitative and quantitative profiles of transcripts from each allele, would allow more precise classification of *TP53* variants and ultimately improve risk assessment and clinical management for LFS patients.

## Materials and methods

### Distribution and frequencies of *TP53* missense mutations with predicted effect on splicing

We retrieved all possible single nucleotide substitutions in *TP53* exonic sequences from the IARC/NCI *TP53* Database (R21 version, https://tp53.isb-cgc.org/). SpliceAI (v.1.3) [23, 24] was used for in-silico prediction. The SpliceAI raw delta score, defined as the maximum predicted probability that a variant alters splicing at any position within a ± 10 kb window (recommended for optimal performance), was recorded for each substitution. A delta score threshold of ≥0.4 was applied, as this value provided a balance between sensitivity (97.9%) and specificity (72.4%) in receiver operating characteristic (ROC) analysis (AUC = 0.95) using a publicly available validation webtool (https://gwiggins.shinyapps.io/lr_shiny/, standard settings), based on an external validation dataset comprising more than 3000 variants across eight disease genes, not *TP53* specific, developed by Walker et al. [25], with threshold selection consistent with approaches described in Wu et al. [26].

Frequencies of missense *TP53* mutations with a SpliceAI delta score ≥0.4 have been analyzed in three different datasets: (1) germline *TP53* variants (IARC/NCI *TP53* Database, https://tp53.isb-cgc.org/; R21 version), (2) cancer–related somatic *TP53* variants (COSMIC database; downloaded on November 12, 2024; https://cancer.sanger.ac.uk/) and (3) non-cancer related *TP53* germline variants (gnomAD, v. 4.1.0, https://gnomad.broadinstitute.org/).

### Functional impact of predicted SE-SNVs

Missense variants were assigned to functional Classes A–D according to the classification proposed by P. Hainaut and collaborators [5], which exploited the yeast-based transactivation assay dataset developed by C. Ishioka and collaborators [15]. We also integrated data from a CRISPR–Cas9–mediated homology-directed repair saturation mutagenesis screen [9], which assessed the proliferative fitness of *TP53* variants in the DNA-binding domain (exons 5–8) following p53 pathway activation by MDM2 inhibition. Enrichment scores from this assay were normalized to relative fitness scores (rfs), ranging from −1 (synonymous mutations) to +1 (nonsense mutations). For each variant, rfs values were correlated with the corresponding SpliceAI delta scores using Pearson regression (ggplot2 and ggpubr R packages, version 2023.12.1 + 402). Variants included in the rfs correlation analysis were missense SNVs in exons 5–8 (the region covered by the Funk et al. [9] saturation mutagenesis dataset; *n* = 110 variants with available rfs scores) with raw SpliceAI delta scores ≥0.1 (to exclude negligible predicted splice effects).

### Minigene splicing assay

We used the exon trapping vector pSPL3 [27] according to the modified protocol of Tompson and Young [28], with simian green monkey kidney COS1 cells as the recipient cell line. The pSPL3 vector contains a small artificial gene composed of an SV40 promoter, an exon–intron–exon sequence with functional splice donor and acceptor sites, and a late polyadenylation signal. The single intron includes a multiple cloning site that can accommodate a genomic fragment to create a minigene expression construct. We divided the *TP53* cDNA into three fragments (“boxes”): Box 1: exons 2–5 (1701 nt), Box 2: exons 5–8 (1678 nt), and Box 3: exons 8–10 (3,142 nt); each fragment was flanked by 50 intronic nucleotides upstream and downstream of the canonical donor/acceptor splice sites (Supplementary Fig. 2). SE-SNVs with SpliceAI delta scores ≥ 0.75 and located within 60 nt of a splice junction were introduced into these constructs by site-directed mutagenesis. Additional constructs were generated for the synonymous variants p.(Thr125 = ) (c.375 G > A, c.375 G > T, c.375 G > C), p.(Glu224 = ) (c.672 G > A), and p.(Gln331 = ) (c.993 G > A).

All constructs were synthesized and sequence-verified by Azenta Life Sciences – Genewiz (Leipzig, Germany). Plasmids were transformed and amplified in *E. coli* TOP10 (Thermo Fisher, Ref C505003) and purified for transfection. COS1 cells were seeded at 4 × 10 ^5^ cells/well in 6 cm culture dishes and transfected at 70–80% confluency according to the Tompson and Young [28] protocol, with minor modifications. A wild-type *TP53* minigene construct was included as a positive control for canonical splicing. A transfection reaction containing buffer only (no plasmid DNA) was used as a negative control to monitor nonspecific signals. Total RNA was isolated using the NucleoSpin RNA Plus kit (Macherey-Nagel, Düren, Germany; Ref 740990.50). Reverse transcription was performed with the iScript™ Reverse Transcription Supermix kit (Bio-Rad, Providence, RI; Ref 1708840). PCR amplification was carried out using primers V1-F (5′-TCTGAGTCACCTGGACAACC-3′, exon 1) and V2-R (5′-ATCTCAGTGGTATTTGTGAGC-3′, exon 2) with the Platinum SuperFi DNA Polymerase kit (Thermo Fisher, Ref 12351010). PCR products were resolved by agarose gel electrophoresis, purified, and analyzed by Sanger sequencing (GENEWIZ from Azenta Life Sciences, Leipzig, Germany).

### TCGA RNA-Seq analysis

For SE-SNVs tested in the minigene assay, corresponding mRNA expression data were retrieved, when available, from the TCGA RNA-Seq datasets (https://www.cancer.gov/ccg/access-data) and compared with expression levels of wild-type *TP53*. RNA-Seq level-3 data from 33 cancer cohorts were downloaded from Firebrowse (http://firebrowse.org), and raw read counts were normalized across the combined dataset using the DESeq2 package (v1.46.0), excluding genes with fewer than 10 total read counts across all samples. mRNA expression levels for each patient were matched to their mutation profiles obtained from cBioPortal (https://www.cbioportal.org/). Differences in *TP53* expression between mutant and wild-type samples were calculated as the percentage change (reduction or increase) relative to wild-type *TP53* expression levels. To examine splicing patterns, raw RNA-Seq data (NCI dbGaP Study Accession: phs000178.v11.p8) were analyzed, and splice junction usage was visualized using Sashimi plots generated in R with the ggsashimi tool [29].

### *TP53* germline variant dataset

Clinical information for carriers of Class C/D SE-SNVs was compiled from the germline *TP53* variants database (https://tp53.cancer.gov/), an extended literature search, and LFS registries in France and Germany [2, 18]. Carriers of missense variants from Class A–D and of the splice variant c.375 G > A (p.(Thr125 = )), were identified from the germline *TP53* variants database. Individuals harboring multiple *TP53* variants were excluded because the contribution of each variant could not be reliably determined. Carriers of the Brazilian founder variant *TP53* p.(Arg337His) who were not recruited through a family history of cancer were also excluded to avoid analytical bias.

### Genotype–phenotype correlations

Lifetime cancer incidence by variant class was assessed using either the age at first cancer diagnosis or the cancer-free age of individuals, by fitting Kaplan–Meier curves. Median age at first cancer and corresponding 95% confidence intervals (CIs) were extracted from the Kaplan–Meier curves using the Brookmeyer–Crowley method. The age distribution of all cancers, including both primary and secondary malignancies, was analyzed. Tumor distribution within each class was determined based on topography, with organ groups relevant to LFS (adrenal gland, brain, bone, soft tissue, breast, hematopoietic system, and other organs) consolidated for analysis. All analyses were performed in R.

### Nomenclature

We describe all *TP53* variants according to HGVS recommendations, with cDNA numbering based on the reference transcript NM_000546.6; genomic coordinates (g.) are given on GRCh38 using chromosome 17 reference sequence NC_000017.11. For synonymous variants we use HGVS-compliant notation (e.g., p.(Thr125 = )).

## Supplementary information

Supplementary information

Supplementary table 2

Supplementary table 3

## Data Availability

All data generated or analyzed during this study are included in this article and its supplementary information files. Source data for all figures and tables are available from the corresponding author upon request. Publicly available RNA-seq datasets from *The Cancer Genome Atlas* (TCGA) were used in this study; accession numbers and variant details are provided in the Supplementary Information.
