# Supplementary material for: Tumor patterns and cancer risk in carriers of TP53 exonic germline variants that alter mRNA splicing
Source: Eur J Hum Genet. 2026 Mar 3;34(8):1112–20. doi: 10.1038/s41431-026-02061-6 (PMC13424335; doi:10.1038/s41431-026-02061-6)
Supplement: Supplementary file 1 — Supplementary information [file 41431_2026_2061_MOESM1_ESM.pdf]

Supplementary Information

Tumor Patterns and Cancer Risk in Carriers of *TP53* exonic Germline Variants that alter mRNA Splicing

Deborah Schoenegger[1], Emilie Montellier[1], Sandrine Blanchet[1], Claire Freycon[2], Paola Monti[3], Catherine Goudie[4], Gaëlle Bougeard[5], Christian P. Kratz[6], Pierre Hainaut[1*†], Anna Reymer[1*]

[1]University Grenoble Alpes, Inserm 1209, CNRS 5309, Institute for Advanced Biosciences, F38000 Grenoble, France

[2]Department of Pediatric Hematology-Oncology, Grenoble Alpes University Hospital, Grenoble, France

[3]Neuro-oncology and Mutagenesis, IRCCS Azienda Ospedaliera Metropolitana, 16132 Genoa, Italy

[4]Department of Pediatrics, Division of Hematology-Oncology, Montreal Children's Hospital, McGill University Health Centre, Montreal, QC, Canada

[5]University Rouen Normandie, Inserm U1245, Normandie Univ, CHU Rouen, Department of Genetics, F-76000 Rouen, France

[6]Pediatric Hematology and Oncology, Hannover Medical School, Hannover, Germany

[†] Deceased

*: co-senior authors, correspondence to: Anna.Reymer@univ-grenoble-alpes.fr

Table of Content:

SI Table 1. Sources of SE-SNV carriers included in the genotype–phenotype analyses, compiled from IARC/NCI, systematic literature review, and LFS registries. — p. 2

SI Figure 1. Receiver Operating Characteristic (ROC) curve evaluating SpliceAI performance in predicting splice-disruptive variants. — p. 3

SI Tables 2 and 3: attached as a separate Excel file.

SI Figure 2. *TP53* minigene constructs expressed in COS-1 cells, including schematic representation and RT-PCR analysis. — p. 4

SI Figure 3. Sanger sequencing electropherograms of minigene assay results with indications of cryptic splice site activation. — p. 5-6

SI Figure 4. Sashimi plots showing raw RNA-Seq read alignments from TCGA patients carrying selected *TP53* SE-SNVs (p.(Ser106Arg), p.(Thr125=), p.(Gln331=)) alongside normal tissue controls. — p. 7-9

SI Figure 5. Density plots showing the age distribution of all cancer diagnoses for each variant category. — p. 10

SI Figure 6. Distribution of cancer types among carriers of missense variants (Class A–D), spliceogenic Class C/D variants, intronic splice variants, and the synonymous mutation p.(Thr125=). — p. 11

SI Table 4. Number of carriers for each category analyzed in this study, and the corresponding proportions. — p. 12

References. — p. 13

SI Table 1: Sources of SE-SNV carriers Included in the genotype–phenotype analyses.

Database/Source	SE-SNV	ProtDescription	YTA_class[3]	Reference
NCI/IARC Germline	c.318C>G	p.(Ser106Arg)	C	PMID: 11518751
NCI/IARC Germline	c.672G>T	p.(Glu224Asp)	D	PMID: 19930417
NCI/IARC Germline	c.356C>G	p.(Ala119Gly)	C	PMID: 30076369
Literature	c.40C>G	p.(Leu14Val)	D	PMID: 26189108
Literature	c.559G>C	p.(Gly187Arg)	C	PMID: 30239254
Literature	c.559G>A	p.(Gly187Ser)	C	PMID: 34771502 and 39962599
LFS registries	c.559G>A	p.(Gly187Ser)	C	LFS registry (France)[4]
LFS registries	c.671A>C	p.(Glu224Ala)	D	LFS registry (France)[4]
LFS registries	c.559G>C	p.(Ser261Ile)	D	LFS registry (France)[4]
LFS registries	c.356C>G	p.(Ala119Gly)	C	LFS registry (Germany)[5]

The table documents the provenance of the SE-SNV cases analyzed in this study. Cases were compiled from three sources: (i) the IARC/NCI germline *TP53* database[6] (version 20), (ii) a systematic literature review conducted between 2019–2025, and (iii) the French[4] and German[5] Li-Fraumeni syndrome (LFS) clinical registries. Duplicate entries across datasets were removed. This compilation is provided to ensure transparency of case selection and traceability of references.

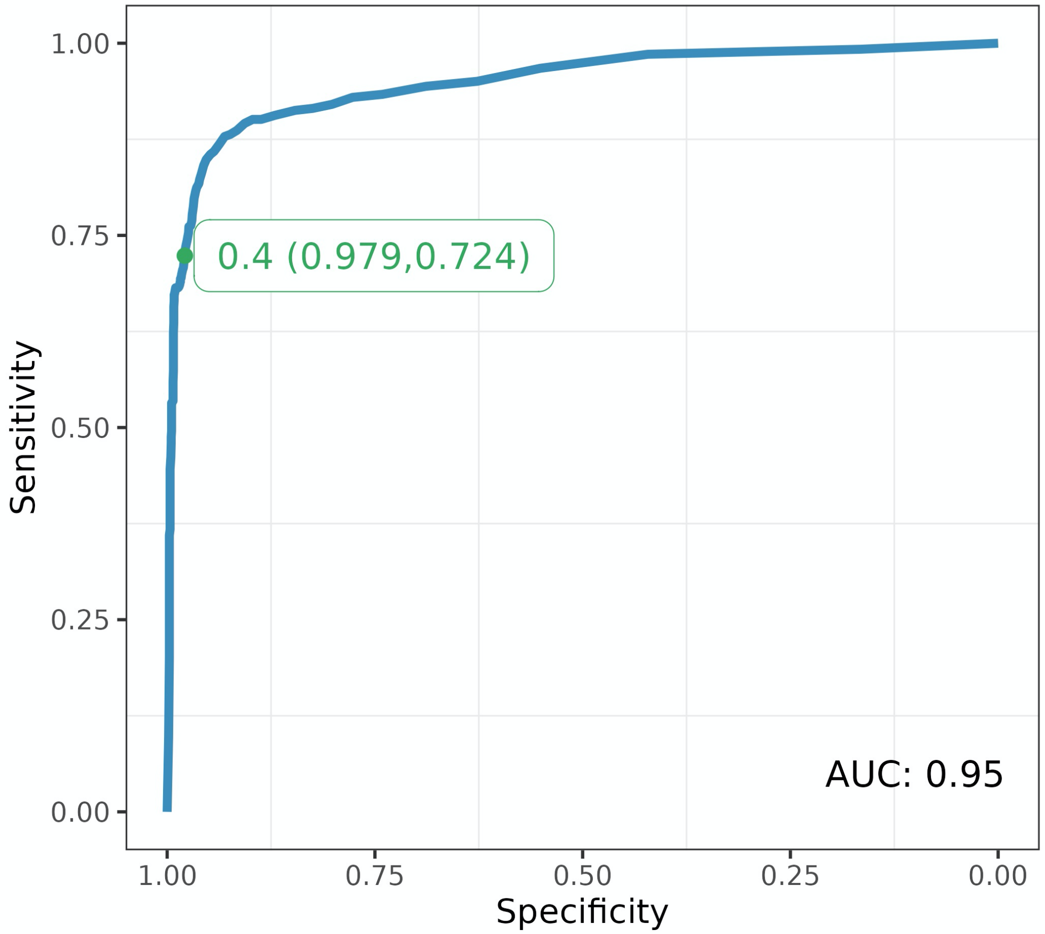

SI Figure 1: Receiver Operating Characteristic (ROC) curve evaluating SpliceAI performance in predicting splice-disruptive variants. The curve illustrates the trade-off between sensitivity and specificity across varying SpliceAI score thresholds. A threshold of 0.4 is highlighted, yielding a sensitivity of 0.979 and a specificity of 0.724. The area under the curve (AUC) is 0.95, indicating strong predictive performance. This analysis is based on a curated dataset of 3,021 variants across BRCA1/2, mismatch repair genes (*MLH1, MSH2, MSH6, PMS2*), *NF1*, and *POU1F1*, which includes *in vitro* splicing assay results[1].

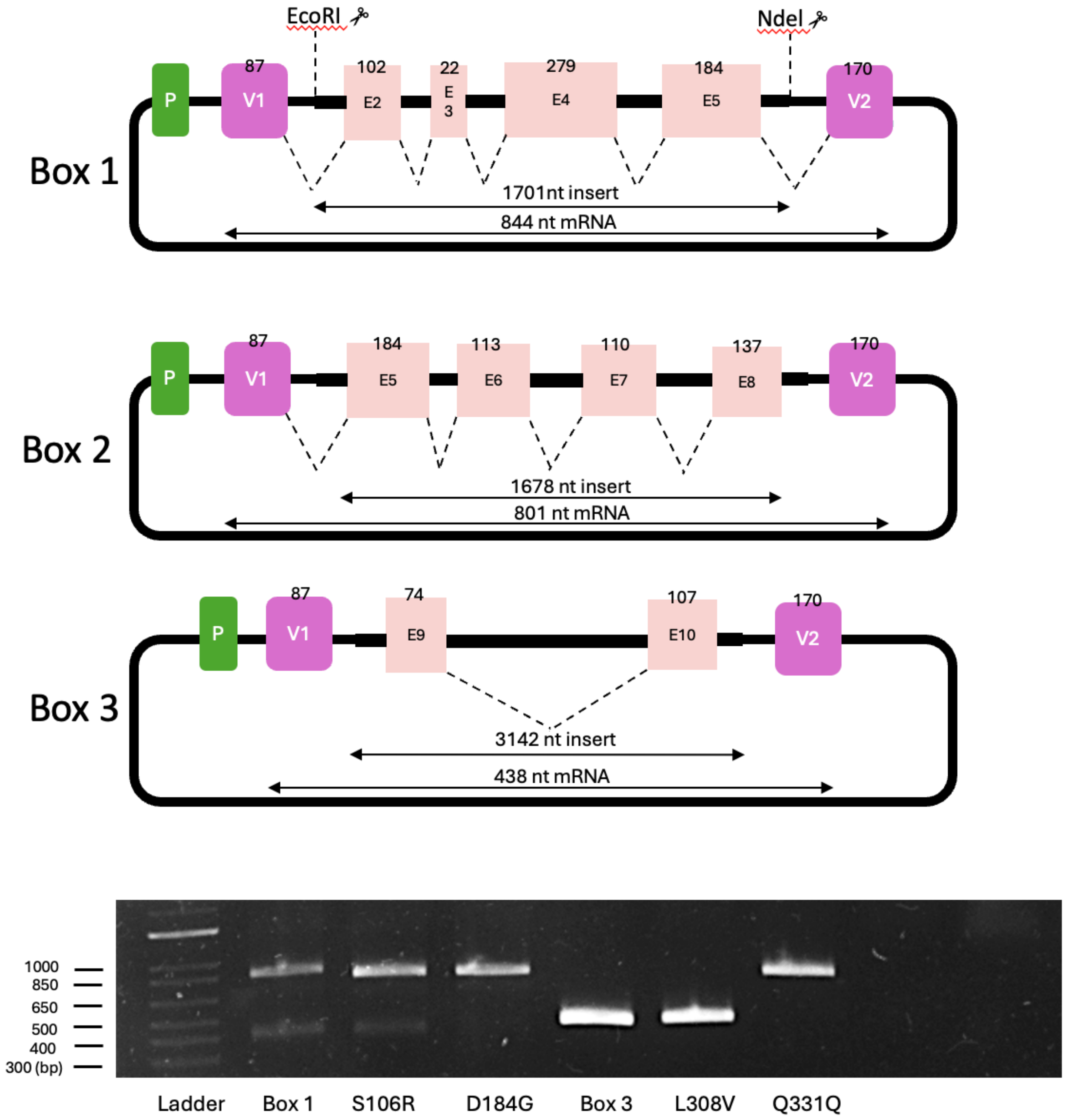

SI Figure 2: *TP53* minigene constructs expressed in COS-1 cells. Genomic regions of the *TP53* gene were divided into three overlapping fragments ("Box 1," "Box 2," and "Box 3") and cloned into the pSPL3 exon trapping vector, which contains vector-specific exons V1 and V2 and a promoter (P). Each construct contains selected *TP53* exons (E2–E10) and flanking intronic sequences, indicated by exon numbers and dashed lines. Arrows indicate the expected size of the inserted genomic DNA (nt insert) and the spliced mRNA (nt mRNA) following expression in COS-1 cells.

The lower panel shows an example of RT-PCR analysis on RNA extracted from COS-1 cells transfected with wild-type (Box1 and Box3) or mutant minigene constructs. PCR products were visualized in agarose gel and PCR products were Sanger sequenced to verify alternative splicing patterns.

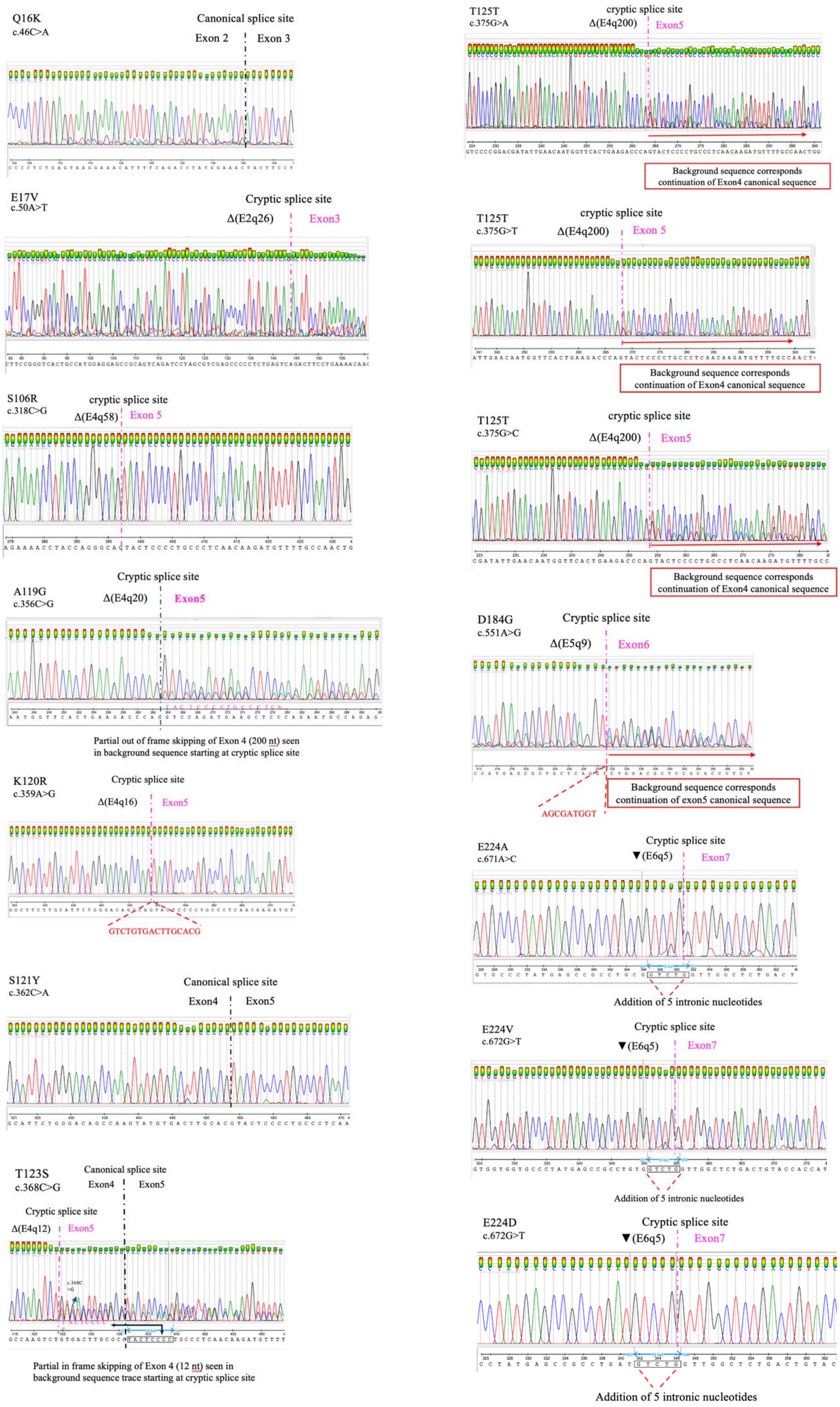

Q16K
c.46C>A
Canonical splice site
Exon 2
Exon 3
E17V
c.50A>T
Cryptic splice site
Δ(E2q26)
Exon3
S106R
c.318C>G
cryptic splice site
Δ(E4q58)
Exon 5
A119G
c.356C>G
Cryptic splice site
Δ(E4q20)
Exon5
Partial out of frame skipping of Exon 4 (200 nt) seen in background sequence starting at cryptic splice site
K120R
c.359A>G
Cryptic splice site
Δ(E4q16)
Exon5
GTCTGTGACTTGCACG
S121Y
c.362C>A
Canonical splice site
Exon4
Exon5
T123S
c.368C>G
Canonical splice site
Exon4
Exon5
Cryptic splice site
Δ(E4q12)
Exon5
Partial in frame skipping of Exon 4 (12 nt) seen in background sequence trace starting at cryptic splice site
T125T
c.375G>A
cryptic splice site
Δ(E4q200)
Exon5
Background sequence corresponds continuation of Exon4 canonical sequence
T125T
c.375G>T
cryptic splice site
Δ(E4q200)
Exon 5
Background sequence corresponds continuation of Exon4 canonical sequence
T125T
c.375G>C
cryptic splice site
Δ(E4q200)
Exon5
Background sequence corresponds continuation of Exon4 canonical sequence
D184G
c.551A>G
Cryptic splice site
Δ(E5q9)
Exon6
Background sequence corresponds continuation of exon5 canonical sequence
AGCGATGGT
E224A
c.671A>C
Cryptic splice site
▼(E6q5)
Exon7
Addition of 5 intronic nucleotides
E224V
c.672G>T
Cryptic splice site
▼(E6q5)
Exon7
Addition of 5 intronic nucleotides
E224D
c.672G>T
Cryptic splice site
▼(E6q5)
Exon7
Addition of 5 intronic nucleotides

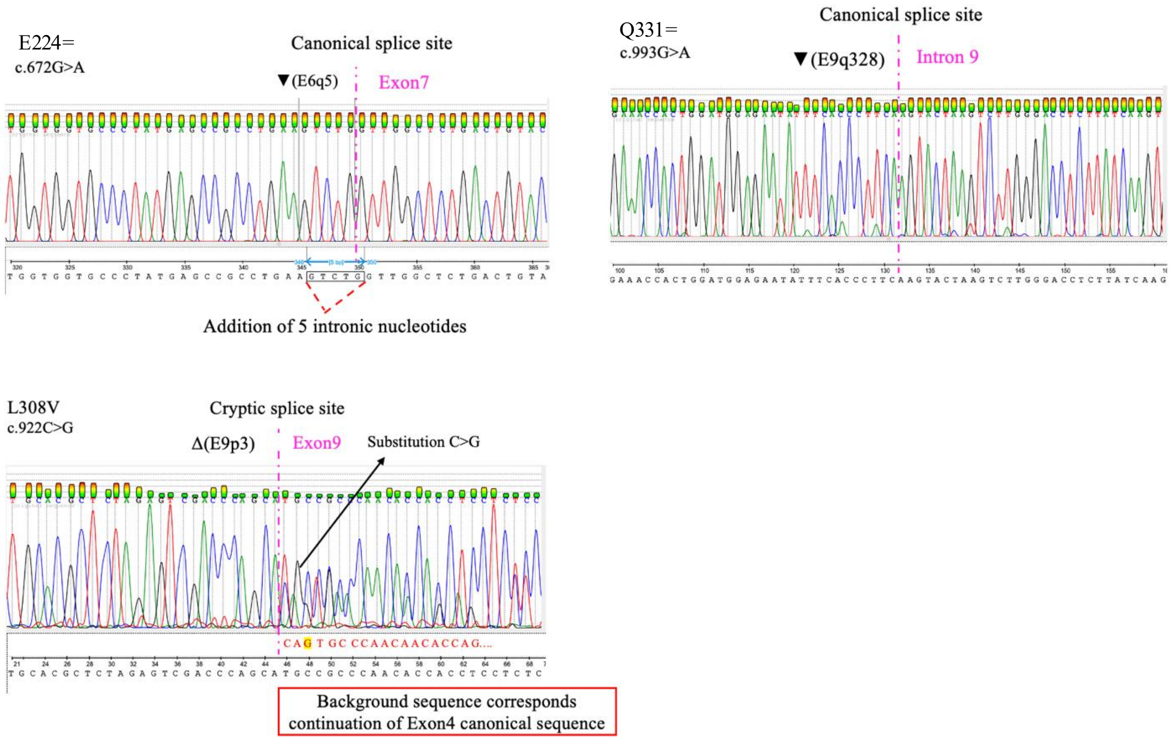

SI Figure 3: Sanger sequencing electropherograms of minigene assay results with indications of locations of cryptic splice sites.

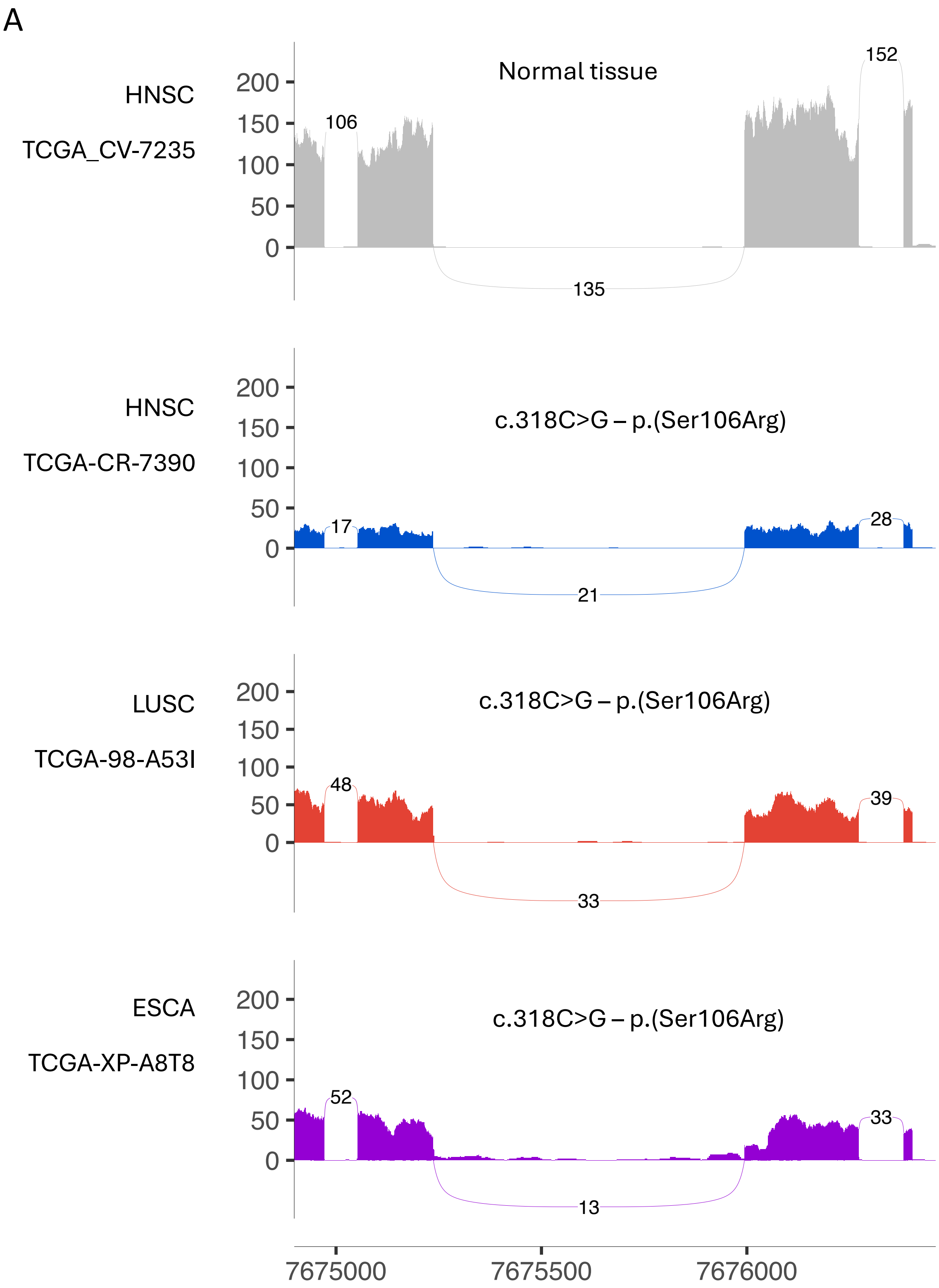
A
HNSC
TCGA_CV-7235
Normal tissue
106
152
135
HNSC
TCGA-CR-7390
c.318C>G – p.(Ser106Arg)
17
28
21
LUSC
TCGA-98-A53I
c.318C>G – p.(Ser106Arg)
48
39
33
ESCA
TCGA-XP-A8T8
c.318C>G – p.(Ser106Arg)
52
33
13
200
150
100
50
0
7675000
7675500
7676000

B

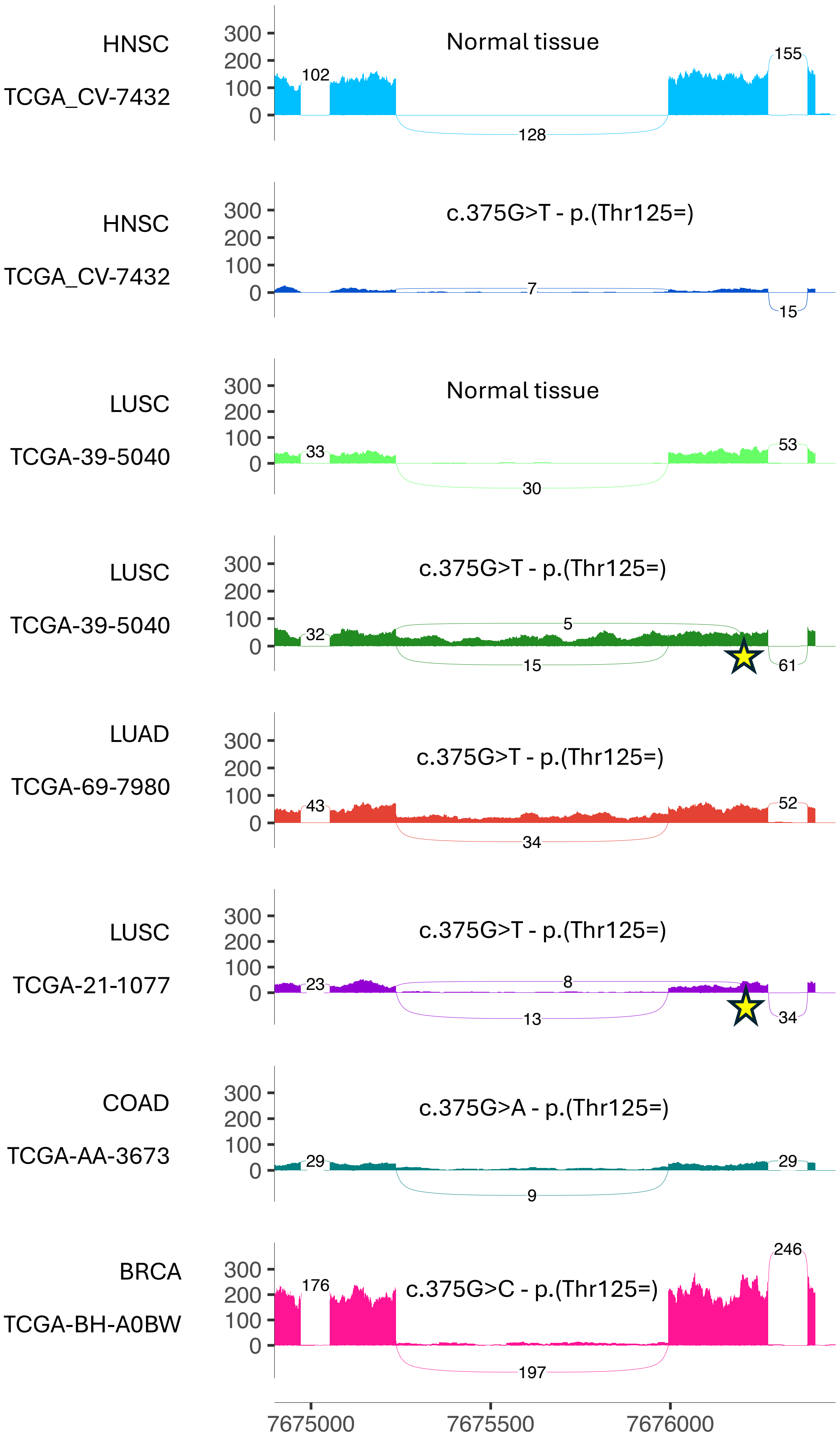

HNSC
TCGA_CV-7432
Normal tissue
102
155
128
HNSC
TCGA_CV-7432
c.375G>T - p.(Thr125=)
7
15
LUSC
TCGA-39-5040
Normal tissue
33
53
30
LUSC
TCGA-39-5040
c.375G>T - p.(Thr125=)
32
5
15
61
LUAD
TCGA-69-7980
c.375G>T - p.(Thr125=)
43
52
34
LUSC
TCGA-21-1077
c.375G>T - p.(Thr125=)
23
8
13
34
COAD
TCGA-AA-3673
c.375G>A - p.(Thr125=)
29
29
9
BRCA
TCGA-BH-A0BW
c.375G>C - p.(Thr125=)
176
246
197
300
200
100
0
7675000
7675500
7676000

C

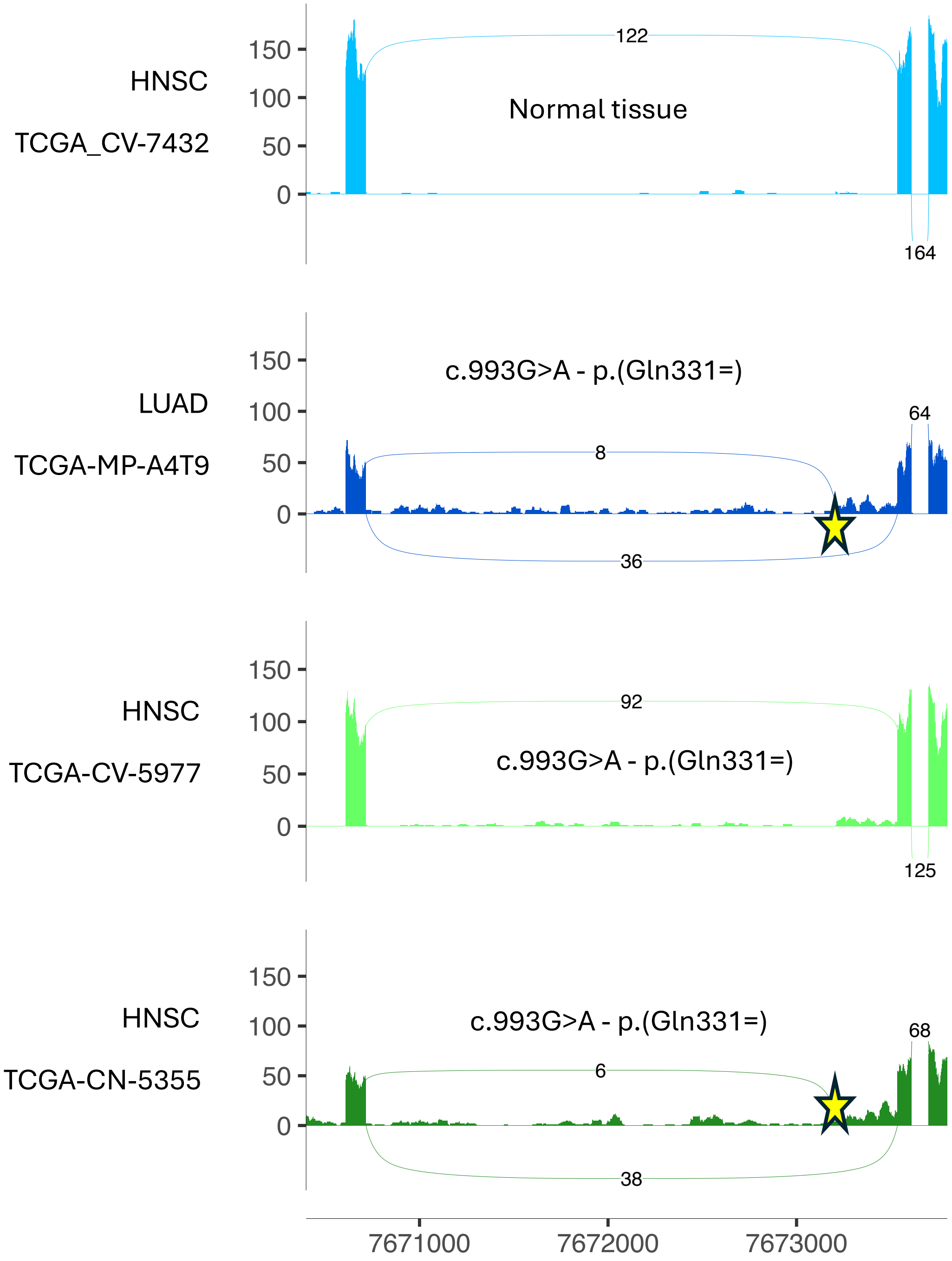

SI Figure 4: Sashimi plots showing raw read alignments from TCGA patients with **A**: p.(Ser106Arg) *TP53* variant (c.318C>G); **B**: p.(Thr125=) (c.375G>T, c.375G>A and c.375G>C); and **C:** p.(Gln331=) (c.993G>A) mutations together with a normal tissue sample in each case as comparison. Sashimi plots of variants at codon 224, namely p.(Glu224Asp) (c.672G>C or c.672G>T) and p.(Glu224=) (c.672G>A) are shown in Velkova et al.[2] LUSC, lung squamous cell carcinoma; LUAD, lung adenocarcinoma; HNSC, head and neck squamous cell carcinoma; COAD, colon adenocarcinoma; ESCA, esophageal carcinoma; BRCA, breast cancer.

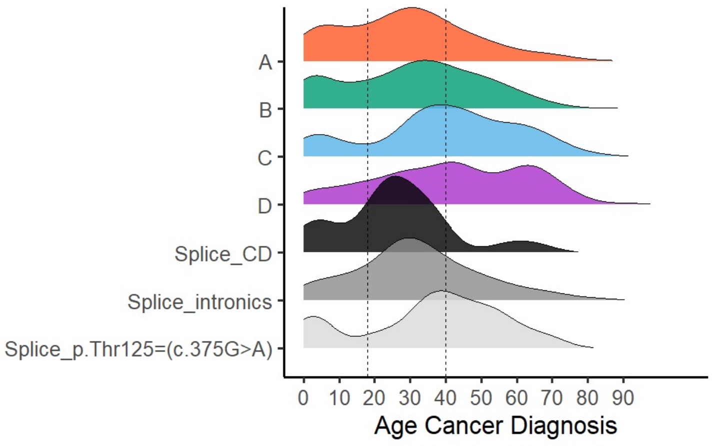

SI Figure 5: Density plots showing the age distribution of all cancer diagnoses for each variant category.

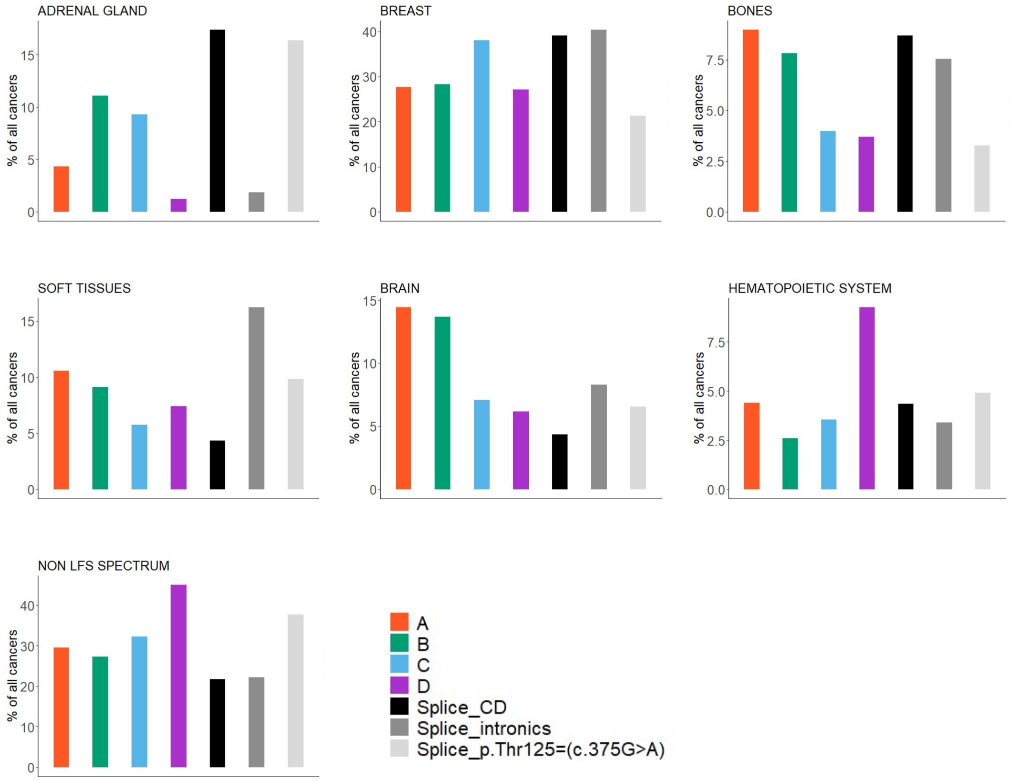

SI Figure 6: Distribution of cancer types for missense *TP53* variants of class A-D, class C and D missense mutations with predicted spliceogenic effects (25 cancers in total), intronic splice mutations and the synonymous mutation p.(Thr125=) (c.375G>A).

SI Table 4: Number of carriers for each category analysed in the current study, and the corresponding proportions.

Category/Class	Number of individuals	Proportion
A	1426	59%
B	290	12%
C	238	10%
D	171	7%
Splice_CD* (Splice_C + Splice_D)	18 (12 + 6)	1%
Splice_intronics	219	9%
Splice_p.Thr125=(c.375G>A)	59	2%
TOTAL	2421	100%

*Proportion of CD_splice variants relative to the total number of individuals in Class C and D *TP53* variants according to Montellier et al.[3]:

C = 238 | **Splice_C** = 12 (**4.8% of class C** individuals)

D = 171 | **Splice_D** = 6 (**3.4% of class D** individuals)

Total C+D = 409 | Total **Splice_CD** = 18 (**4.2% of class C+D**)

References

1. Walker, L.C., Hoya, M. de la, Wiggins, G.A.R., Lindy, A., Vincent, L.M., Parsons, M.T., Canson, D.M., Bis-Brewer, D., Cass, A., Tchourbanov, A., et al. (2023). Using the ACMG/AMP framework to capture evidence related to predicted and observed impact on splicing: Recommendations from the ClinGen SVI Splicing Subgroup. Am. J. Hum. Genet. *110*, 1046–1067. https://doi.org/10.1016/j.ajhg.2023.06.002.

2. Velkova, I., Cappato, S., Rivera, D., Romano, F., Schonneger, D., Bocciardi, R., Hainaut, P., De Marco, P., Gismondi, V., Cirmena, G., et al. (2025). Missense but mis-spliced: germline TP53 variant c.671A>C (p.E224A) and the path from uncertainty to pathogenicity at medRxiv, https://doi.org/10.1101/2025.07.31.25332437 https://doi.org/10.1101/2025.07.31.25332437.

3. Montellier, E., Lemonnier, N., Penkert, J., Freycon, C., Blanchet, S., Amadou, A., Chuffart, F., Fischer, N.W., Achatz, M.-I., Levine, A.J., et al. (2024). Clustering of TP53 variants into functional classes correlates with cancer risk and identifies different phenotypes of Li-Fraumeni syndrome. iScience *27*, 111296. https://doi.org/10.1016/j.isci.2024.111296.

4. Bougeard, G., Renaux-Petel, M., Flaman, J.-M., Charbonnier, C., Fermey, P., Belotti, M., Gauthier-Villars, M., Stoppa-Lyonnet, D., Consolino, E., Brugières, L., et al. (2015). Revisiting Li-Fraumeni Syndrome From TP53 Mutation Carriers. J. Clin. Oncol. *33*, 2345–2352. https://doi.org/10.1200/JCO.2014.59.5728.

5. Penkert, J., Strüwe, F.J., Dutzmann, C.M., Doergeloh, B.B., Montellier, E., Freycon, C., Keymling, M., Schlemmer, H.-P., Sänger, B., Hoffmann, B., et al. (2022). Genotype–phenotype associations within the Li-Fraumeni spectrum: a report from the German Registry. J. Hematol. Oncol. *15*, 107. https://doi.org/10.1186/s13045-022-01332-1.

6. de Andrade, K.C., Lee, E.E., Tookmanian, E.M., Kesserwan, C.A., Manfredi, J.J., Hatton, J.N., Loukissas, J.K., Zavadil, J., Zhou, L., Olivier, M., et al. (2022). The TP53 Database: transition from the International Agency for Research on Cancer to the US National Cancer Institute. Cell Death Differ. *29*, 1071–1073. https://doi.org/10.1038/s41418-022-00976-3.
